# Depression and Anxiety Prevention Based on Cognitive Behavioral Therapy for At-Risk Adolescents: A Meta-Analytic Review

**DOI:** 10.3389/fpsyg.2017.01066

**Published:** 2017-06-28

**Authors:** Sanne P. A. Rasing, Daan H. M. Creemers, Jan M. A. M. Janssens, Ron H. J. Scholte

**Affiliations:** ^1^Behavioural Science Institute, Radboud UniversityNijmegen, Netherlands; ^2^GGZ Oost BrabantBoekel, Netherlands; ^3^PraktikonNijmegen, Netherlands

**Keywords:** prevention, depression, anxiety, cognitive behavioral therapy, adolescents, meta-analysis, indicated, selective

## Abstract

Depression and anxiety disorders are among the most common mental disorders during adolescence. During this life phase, the incidence of these clinical disorders rises dramatically, and even more adolescents suffer from symptoms of depression or anxiety that are just below the clinical threshold. Both clinical and subclinical levels of depression or anxiety symptoms are related to decreased functioning in various areas, such as social and academic functioning. Prevention of depression and anxiety in adolescents is therefore imperative. We conducted a meta-analytic review of the effects of school-based and community-based prevention programs that are based on cognitive behavioral therapy with the primary goal preventing depression, anxiety, or both in high risk adolescents. Articles were obtained by searching databases and hand searching reference lists of relevant articles and reviews. The selection process yielded 32 articles in the meta-analyses. One article reported on two studies and three articles reported on both depression and anxiety. This resulted in a total of 36 studies, 23 on depression and 13 on anxiety. For depression prevention aimed at high risk adolescents, meta-analysis showed a small effect of prevention programs directly after the intervention, but no effect at 3–6 months and at 12 months follow-up. For anxiety prevention aimed at high risk adolescents, no short-term effect was found, nor at 12 months follow-up. Three to six months after the preventive intervention, symptoms of anxiety were significantly decreased. Although effects on depression and anxiety symptoms were small and temporary, current findings cautiously suggest that depression and anxiety prevention programs based on CBT might have small effects on mental health of adolescents. However, it also indicates that there is still much to be gained for prevention programs. Current findings and possibilities for future research are discussed in order to further improve the effectiveness of targeted prevention on internalizing disorders.

## Background

Depression and anxiety are among the most common mental disorders during adolescence (Kessler et al., 2001; Roza et al., 2003), with a prevalence of 5.6% for depression (Costello et al., 2006; Stallard et al., 2012) and a prevalence of 3–20% for anxiety (Albano et al., 2003). Research has shown that among 13–17 year old adolescents the lifetime prevalence is estimated to be 12.6% for depression and 32.4% for anxiety disorders (Kessler et al., 2012). Even more adolescents suffer from subclinical levels of depression or anxiety, with 21.4% of the adolescents estimated to suffer from subclinical depression symptoms (Smit et al., 2003). Unfortunately, the number of adolescents suffering from subclinical anxiety is unknown.

Depression and anxiety during adolescence are associated with decreased psychosocial functioning (Birmaher et al., 1996), that is, malfunctioning in social relations (Strauss et al., 1987), poor academic performance or school drop-out (Strauss et al., 1987; Birmaher et al., 1996), and an increased risk for substance abuse, other mental health problems, and suicide (Birmaher et al., 1996). Further, adolescents with a depression or anxiety disorder are at considerable risk for developing recurrent depression and anxiety disorders later in life (Pine et al., 1999; Aalto-Setala et al., 2002; Copeland et al., 2014). These negative consequences are comparable between adolescents who meet the criteria for a depression or anxiety disorder and adolescents with subclinical depression and anxiety symptoms (Lewinsohn et al., 2000; Aalto-Setala et al., 2002; Beesdo et al., 2009). Therefore, it is imperative to reduce the incidence of depression and anxiety, but also to prevent further development of depression and anxiety symptoms. Because depression and anxiety symptoms rise dramatically during adolescence, this seems to be the appropriate age to implement prevention, because the risk for depression and anxiety rises during this phase. Further, adolescents are, better than younger children, able to understand the concepts that are being taught in the prevention programs due to their improved reasoning (Hankin et al., 1998; Stice et al., 2009).

Several prevention programs have been developed to prevent depression and anxiety during adolescence. These programs utilize different types of prevention strategies and focus on populations with different risks of developing depression or anxiety (Mrazek and Haggerty, 1994). First, universal prevention programs are intended for all individuals in a population, regardless of their level of risk. These programs have shown mixed results in reducing and preventing depression and anxiety symptoms (Horowitz and Garber, 2006; Sheffield et al., 2006; Fisak et al., 2011; Teubert and Pinquart, 2011; Merry et al., 2012; Hetrick et al., 2016). Second, selective prevention programs are developed to target populations with risk factors, which are known to be related to the onset of depression and anxiety. Selective prevention programs can be aimed at children of parents with psychopathology or children from lower socio-economical environments (Hyun et al., 2005; Garber et al., 2009; Fisak et al., 2011; Merry et al., 2012; Hetrick et al., 2015). Third, indicated prevention programs are developed to target adolescents who already have elevated symptoms of depression or anxiety, but the symptoms do not qualify for a clinical diagnosis. Results of selective and indicated prevention programs, together also called targeted prevention, have shown to be more promising than universal prevention (Horowitz and Garber, 2006; Stice et al., 2009; Fisak et al., 2011; Merry et al., 2012; Hetrick et al., 2016).

Selective and indicated prevention programs are both aimed at populations with risk factors for depression or anxiety. An important risk factor is parental psychopathology, as children are three times more likely to develop a major depressive disorder and two to seven times more likely to develop an anxiety disorder when their parents suffer from depression or anxiety, respectively (Kashani et al., 1990; Birmaher et al., 1996; Beidel and Turner, 1997; Merikangas et al., 1998; Bijl et al., 2002; Lieb et al., 2002; Van Dorsselaer et al., 2006; Micco et al., 2009).

Another risk factor for the development of adolescent depression and anxiety is the experience of stressful life events during adolescence (Grant et al., 2004; Fox et al., 2010; Auerbach et al., 2012). Studies have shown that increased depressive and anxiety symptoms are often preceded by stress (Ge et al., 1994; Garber et al., 2002), and particularly in girls, stress and depression are closely associated during adolescence (Larson and Ham, 1993; Ge et al., 1994; Rudolph and Hammen, 1999). Further, the existence of subclinical symptoms of depression or anxiety, or undiagnosed clinical levels of these disorders, is a risk factor for the development of a clinical disorder (Clarke et al., 1995; Lowry-Webster et al., 2001; Weissman et al., 2006).

Whereas *prevention* in high risk populations aims to decrease the likelihood of the onset of a depressive or anxiety disorder or decrease in symptoms, *treatment* aims to reduce existing symptoms (Garber and Weersing, 2010). In targeting symptoms, prevention seems to parallel treatment in these goals. As we know from reviews of meta-analyses (Butler et al., 2006; Hofmann et al., 2012), cognitive behavioral therapy demonstrated to be an effective treatment for a wide range of psychological problems, including depression and anxiety. Based on the overlap in goals (i.e., decreasing symptoms) between treatment and prevention, techniques of the cognitive behavioral approach seem to be suitable techniques to use in the prevention of depression and anxiety in high-risk adolescents. Although, several prevention programs for depression and anxiety are based on cognitive behavioral theories, to our knowledge, the effects of depression and anxiety prevention programs with this same theoretical background for high-risk adolescents were never reviewed or studied in a meta-analysis. In this meta-analysis, we examined whether prevention programs based on the cognitive behavioral approach are effective in preventing depression and anxiety in high-risk adolescents.

Several reviews and meta-analyses have been conducted to evaluate the effectiveness of depression prevention programs and anxiety prevention programs for adolescents (Neil and Christensen, 2009; Calear and Christensen, 2010; Christensen et al., 2010; Teubert and Pinquart, 2011; Merry et al., 2012). These meta-analyses were focused on either depression or anxiety, and on prevention in general and not on high risk populations. In contrast to those, the present study focused specifically on depression and anxiety prevention programs based on cognitive behavioral therapy approaches for adolescents with a high risk on developing depression or anxiety.

This review intended to identify and describe school-based and community-based prevention programs based on cognitive behavioral therapy with a primary goal of preventing depression, anxiety, or both in adolescents at risk for developing these disorders. Furthermore, we aimed to determine their effectiveness in reducing symptoms of depression and anxiety in the short-term and in the long-term.

## Method

The study design will be reported in accordance with the PRISMA Statement for Reporting Systematic Reviews and Meta-Analyses of Studies That Evaluate Health Care Interventions (Liberati et al., 2009).

### Search and screening

Databases Medline (from 1946), PsycInfo (from 1906), Embase (from 1974), and Eric (from 1965) were electronically searched in July 2013. This searched was updated in February 2017. The key search terms “adolescen^*^ OR teen-age^*^ OR youth^*^,” “prevent^*^ OR early intervent^*^,” “depress^*^ OR anx^*^ OR mood OR internali#ing” AND “at risk OR high risk” were used.

**Figure 1:**
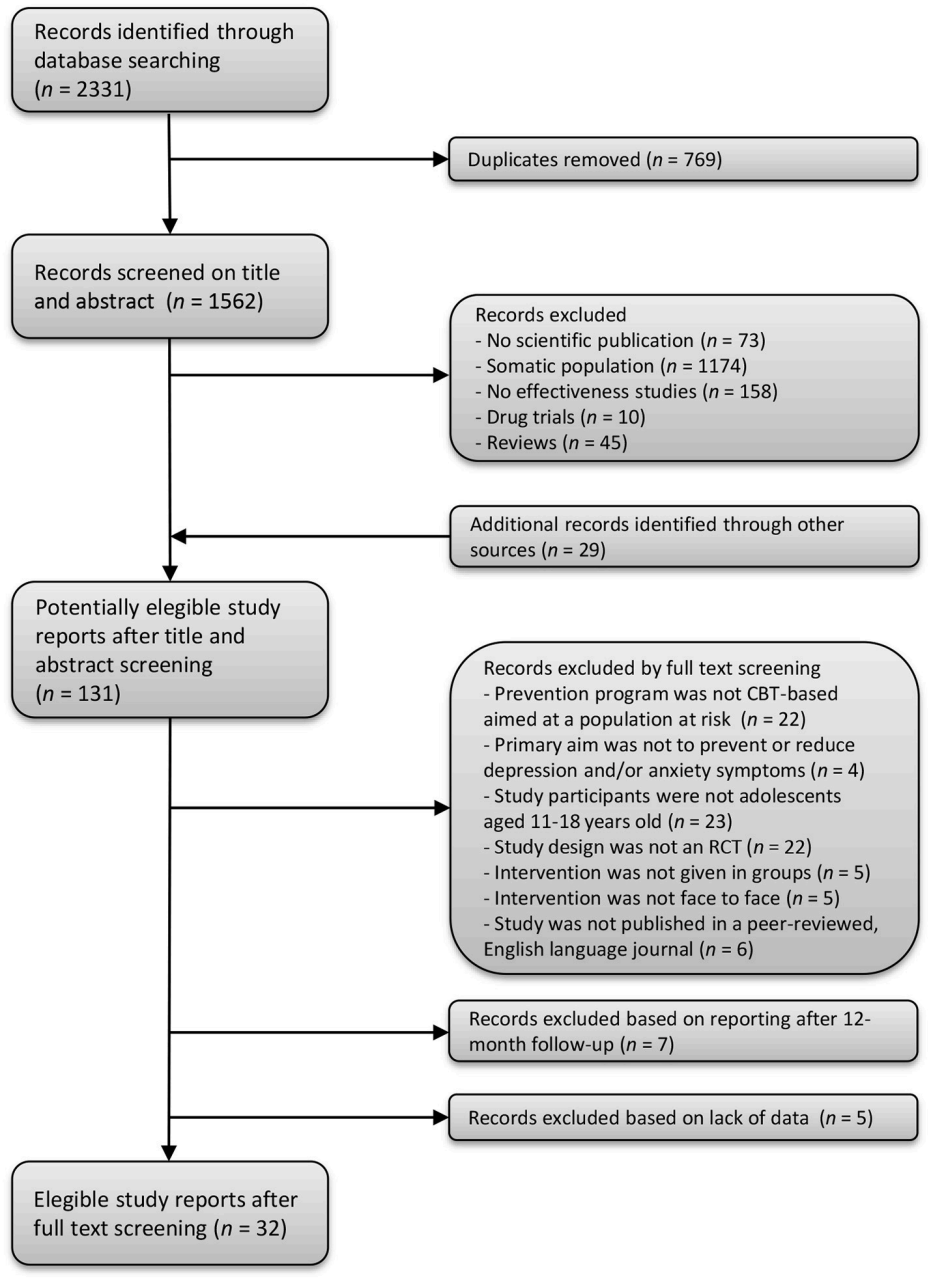
Flow chart of the search strategy and selection of study reports.

Using these specified terms, we identified 2,331 articles. After removing 769 duplicates, 1,562 articles remained. First, titles and abstracts of the 1,562 remaining articles were screened by the first author to determine their relevance to the review. This resulted in the exclusion of 1,460 articles (1,174 articles included populations with other (mental) health problems than depression and/or anxiety; 45 articles were reviews, systematic reviews or meta-analyses; 10 articles reported on drug trials; 158 articles reported on other aspects than effectiveness; and 73 documents were not peer reviewed articles); thus 102 articles remained. Additionally, 29 articles were obtained by hand searching reference lists of relevant articles and reviews. Figure 1 demonstrates the flow chart of selected articles.

Second, of the 131 articles that were identified, the title, abstract and method section of each article were systematically reviewed and considered for inclusion. The inclusion criteria for the current review were that (1) the prevention program was CBT-based and targeted a population at risk (i.e., the program was selective or indicated prevention), (2) the reported intervention aimed to prevent or reduce depression and/or anxiety symptoms, (3) study participants included adolescents aged 11–18, (4) the study design was a randomized controlled trial, (5) the intervention was given in groups, (6) the intervention was delivered face to face, and (7) the study was published in a peer-reviewed, English language journal. Rating the articles was done by five independent reviewers (two postgraduates, one post-doc and two full professors) in which each article was handled by two reviewers. Agreement between raters was between 79 and 96 % (Cohen's Kappa = 0.58–0.92). Differences between reviewers were resolved by consensus. Main reasons for excluding articles can be found in Figure 1. Finally during data extraction, we learned that seven articles reported no measurements on depression or anxiety at baseline, 3 to 6- or 12-month follow-up but only longer duration of follow-up and five articles showed a lack of data, such as missing mean scores or standard deviations in order to calculate effect sizes, and these were also excluded. This resulted in 32 articles included in the meta-analyses.

### Coding of studies

All studies were coded for type of prevention, sample, number of participants, age of participants, percentage of females, group size, number and duration of sessions, characteristics of control condition, randomization, outcome measure, means and standard deviations at post-intervention, means and standard deviations at 6 months follow-up, and means and standard deviations at 12 months follow-up. Coding was done by two independent raters (both postgraduates). Overall agreement between raters was 95%. Differences between raters were resolved through discussion which led to total agreement.

### Statistical analysis

We used the Cochrane Collaboration software Review Manager (RevMan version 5.3) to conduct meta-analyses. We analyzed the data with a random effects model, calculating the standardized mean differences (SMDs), also known as effect size Cohen's *d* (Cohen, 1977), and 95% confidence intervals (CIs). Cohen's effect sizes are generally categorized as small (0.2–0.5), moderate (0.5–0.8), or large (larger than 0.8). We based the effects sizes on the change in self-reported depression scores and anxiety scores. Heterogeneity between trials was assessed using Tau^2^ (estimated standard deviation of underlying effects across studies), Chi^2^ (whether observed differences in results are compatible with chance alone), and *I*^2^ statistic (in which higher values indicate higher heterogeneity), which is defined by the percentage of total variation across trials due to heterogeneity rather than chance (Higgins and Thompson, 2002).

## Results

### Sample characteristics

The sample consisted of 32 articles. One article reported on two studies (Barrett et al., 2005), and three articles reported both on depression as well as anxiety (Gillham et al., 2006b; Manassis et al., 2010). This resulted in 36 studies, 23 on depression and 13 on anxiety.

In the depression study sample (see Table 1), 19 studies used indicated prevention, two used selective prevention, and two used a combination of selective and indicated prevention. Nineteen studies were conducted in schools, four were community-based. The size of the intervention groups varied from small groups of 3–15 adolescents in 16 studies, to large groups (school classes) in three studies. Four studies did not report group size. Intervention intensity ranged from four sessions of 60 min (total of 240 min) to 12 sessions of 120 min (total of 1,440 min). The control conditions were non-intervention groups (i.e., adolescents received no care or guidance at all) in seven studies, usual care groups (i.e., adolescents received personal care or guidance if requested) in 11 studies, waiting list groups (i.e., adolescents received the intervention after completion) in two studies, and active control conditions in three studies (Let's talk program, an educational brochure, and activity groups). In three studies study (Stallard et al., 2012, 2013; Kindt et al., 2014), the intervention was given in school classes as universal prevention, and afterwards the data for high-risk adolescents were analyzed separately. In the other 20 studies, the intervention was given to selected groups based on features of the participants (i.e., elevated levels of depression).

**Table 1:** Summary of descriptive characteristics of depression prevention programs.

**Study: country**	**Summary of intervention**	**Type of prevention; school- or community-based**	**Sample**	**Mean age; Age range**	**N per group**	**Characteristics of sessions**	**N (intervention/** **control); %** **females; randomization**	**Control group**	**Outcome measure**	**Effect size post-intervention**	**Effect size 3–6 months follow-up**	**Effect size 12 months follow-up**
Clarke et al., 1995: US	Coping with stress course teaches at-risk adolescents cognitive techniques to identify and challenge negative thoughts that may contribute to the development of affective disorders	Indicated; school- based	Children or adolescents with elevated but subdiagnostic depressive symptomatology (CES-D > 24)	15.3; 15–16	6–11	15 sessions; 45 min; three times per week	150 (76/74); 70.0%; participant randomization	Usual care	CES-D	−0.34	0.07^b^	0.01
Clarke et al., 2001: US	Cognitive behavioral program that teaches adolescents cognitive restructuring techniques to identify and challenge negative thoughts, with a focus on beliefs related to having a depressed parent	Indicated; community-based	Adolescents with elevated levels of depressive symptoms (CES-D > 24)	14.6; 13–18	6–11	15 sessions; 60 min; frequency unknown	94 (45/49); 59.6%; participant randomization	Usual care	CES-D	−0.46	^a^	−0.53
Dobson et al., 2010: Canada	Coping with stress program is based on CBT and teaches adolescents how to use cognitive restructuring techniques to identify and challenge negative thoughts	Indicated; school-based	Students with elevated depression symptoms (CES-D > 24), but no current or past clinical depression	15.3; 13–18	12–13	15 sessions; 45 min; frequency unknown	46 (25/21); 69.6%; participant randomization	Active: Let's Talk	CDI	0.13	0.12^b^	^a^
Gaete et al., 2016: Chili	The revised program (YPSA-R) teaches adolescents thought restructuring, problem solving skills and planning skills	Indicated; school-based	Students with elevated depressive symptoms (girls: BDI-II ≥15; boys: BDI-II ≥10)	15.9; 14–19	8–15	8 sessions; 45 min; weekly	342 (229/113); 50.0% school randomization	Non-intervention	BDI-II	^a^	−0.01^b^	^a^
Garber et al., 2009: US	Cognitive behavioral prevention program that teaches adolescents skills to identify and challenge negative thoughts and problem-solving skills	Selective and indicated; community- based	Adolescents with at least one parent with major depressive episode and with subsyndromal depressive symptoms	14.8; 13–17	3–10	14 sessions; 90 min; eight weekly and six monthly	316 (159/157); 58.5%; participant randomization	Usual care	CES-D	−0.30	−0.31^b^	^a^
Gillham et al., 2012: US	Penn Resiliency Program is based on CBT and teaches participants the link between thoughts, feelings and behavior and skills for solving interpersonal problems and coping with stress	Indicated; school-based	Students with high levels of depression (on CDI and/or RADS-2)	Unknown; 10–15	Unknown	10 sessions; 90 min; weekly	266 (137/129); 48.0%; participant randomization	Non-intervention	CDI	−0.26	−0.15^b^	^a^
Gillham et al., 2006a: US	Penn Resiliency Program is based on CBT and teaches participants the link between thoughts, feelings and behavior and skills for solving interpersonal problems and coping with stress	Indicated; community-based	Adolescents with elevated depression scores (CDI > 50th percentile)	Unknown; 11–12	Unknown	12 sessions; 90 min; weekly	271 (147/124); 53.1%; participant randomization	Usual care	CDI	0.02	−0.22^b^	−0.24
Gillham et al., 2006b: US	Penn Resiliency Program is based on CBT and teaches participants the link between thoughts, feelings and behavior and skills for solving interpersonal problems and coping with stress	Indicated; school-based	Students with the highest level of symptoms (on combined CDI and RCMAS Z-scores)	Unknown; 12–13	10–12	8 sessions; 90 min; weekly	44 (22/22); 70.5%; participant randomization	Non-intervention	CDI	−0.09	−0.63^b^	−0.45
Hyun et al., 2005: South Korea	Program bases on CBT teaches adolescents to alter their thought and interpretation of the situation and facilitates the development of the individual's adaptive behavior	Selective; community-based	Male runaway adolescents and residing in a shelter	15.2; Unknown	6–8	8 sessions; 90 min; weekly	32 (16/16); 0%; participant randomization	Non-intervention	BDI	−0.70	^a^	^a^
Kindt et al., 2014: Netherlands	OVK is a CBT based program and teaches adolescents recognizing maladaptive thought, and uses cognitive restructuring and problems solving skills	Selective; school-based	Adolescents from low-income areas in The Netherlands	13.42; 11–16	School classes	16 sessions; 45–50 min; weekly	1343 (667/676); 52.3%; school randomization	Non-intervention	CDI	0.02	−0.12^c^	0.09
Kowalenko, 2005: Australia	ACE program aims to build resilience and increase positive coping in young people using cognitive-behavioral and interpersonal techniques	Indicated; school-based	Students with elevated symptoms of depression (CDI ≥ 18)	14.6; 13–16	8–10	8 sessions; 90 min; weekly	143 (87/56); 65.0%; school randomization	Waiting list	CDI	−0.55	^a^	^a^
Manassis et al., 2010: Canada	Feelings club is a CBT program focused on recognizing and managing negative feelings and maladaptive thoughts using cognitive restructuring	Indicated; school-based	Children with elevated depressive symptoms (MASC or CDI t > 60)	Unknown; 8–12	5–10	12 sessions; 60 min; weekly	148 (78/70); 43.2%; participant randomization	Active: Activity group	CDI	0.01	^a^	−0.19
Poppelaars et al., 2016: Netherlands	The first eight lessons of OVK teach adolescents to recognize their own emotions and cognitions, and learn to change maladaptive cognitions into more adaptive ones	Indicated; school-based	Adolescents with elevated depressive symptoms (RADS ≥ 59).	13.32; 11–16	Unknown	8 sessions; 50 min; weekly	101 (50/51); 100%; participant randomization	Non-intervention	RADS	0.12	0.10^c^	0.09
Puskar et al., 2003: US	Teaching kids to cope aims to prevent depression and to maximize coping by focusing on self-esteem, stress and coping	Selective and indicated; school-based	Students from rural area with elevated symptoms of depression (RADS > 60)	16.0; 14–18	Unknown	10 sessions; 45 min; weekly	89 (46/43); 82.0%; participant randomization within schools	Usual care	RADS	−0.47	−0.49^b^	−0.30
Rohde et al., 2014: US	The CB program taught thought identification/recording and cognitive restructuring and an increased involvement in pleasant activities	Indicated; school-based	Students with elevated self-assessed depressive symptoms	13–19	5–9	6 sessions; 60 min; weekly	250 (126/124); 68%; participant randomization within schools	Educational brochure	K-SADS (16 items)	−0.27	−0.06^c^	^a^
Roberts et al., 2003: Australia	Penn Prevention Program is CBT based and teaches children coping strategies to counteract cognitive distortions and deficiencies	Indicated; school-based	Children with elevated depression symptoms (highest scores on CDI per class)	11.9; 11–13	Small groups	12 sessions; 120 min; weekly	52 (25/27); 49.7%; school randomization	Usual care	CDI	0.02	−0.23^b^	^a^
Sheffield et al., 2006: Australia	The program for preventing depression integrated two major cognitive-behavioral components, namely cognitive restructuring and problem-solving skills training	Indicated; school-based	Students with elevated depressive symptoms (top 20% combined standardized CDI and CES-D scores)	14.3; 13–15	8–10	8 sessions; 90 min; weekly	283 (134/149); 69.0%; school randomization	Non-intervention	CDI	−0.14	0.04^b^	0.08
Singhal et al., 2014: India	Coping Skills Program contains identifying negative thinking, chang-ing to positive thinking, using ABC to challenge negative thoughts, dealing with relationship problems and stress	Indicated; school-based	Adolescents with elevated but subclinical symptoms of depression (CDI and CES-DC)	14.5; 13–18	6–7	8 sessions; 45–50 min; weekly	19 (13/6); 82.0%; school randomization	Usual care	CDI	0.77	−3.31^b^	^a^
Stallard et al., 2013: UK	The key elements of RAP-UK are personal strengths, helpful thinking, keeping calm, problem solving, support networks and keeping the peace	Indicated; school-based	Students with elevated depressive symptoms (SMFQ ≥ 5)	14.4; 12016	School classes	9 sessions (and two boosters; 50–6- min); weekly	680 (392/298); 67.2%; randomization per year group	Usual care	SMFQ	^a^	0.10^c^	0.23
Stallard et al., 2012: UK	The resourceful adolescent program is based on CBT and develops skills such as emotion-regulation, coping mechanisms, and thinking styles to protect against depression	Indicated; school-based	Students classified as at risk based on elevated levels of depression (SMFQ > 5)	14.1; 12–16	School classes	9 sessions; 50–60 min; weekly	767 (393/374); 66.0%; randomization per school year	Usual care	SMFQ	^a^	^a^	0.23
Stice et al., 2007: US	The Blues Group is a CBT based program and uses motivational enhancement exercises, strategic self-presentation, behavioral techniques, and group activities	Indicated; school-based	Students with elevated symptoms of depression (CES-D ≥ 20)	18.4; 15–22	6–10	4 sessions; 60 min; weekly	117 (50/67); 70.0%; articipant randomization	Waiting list	BDI	−0.71	−0.13^b^	^a^
Stice et al., 2010: US	CBT based program for the prevention of depression uses motivational enhancement exercises, strategic self-presentation, behavioral techniques, and group activities	Indicated; school-based	Students with elevated depressive symptoms (CES-D ≥ 20)	15.6; 14–19	3–10	6 sessions; 60 min; weekly	173 (89/84); 56.0%; participant randomization stratified for school and gender	Usual care	BDI	−0.61	−0.53^b^	−0.11
Wijnhoven et al., 2014: Netherlands	In the first eight lessons of OVK, adolescents are taught to recognize their own emotions and cognitions, and learn to change maladaptive cognitions into more adaptive ones	Indicated; school-based	Adolescents with elevated levels of depression (CDI ≥ 16)	13.3; 11–15	11–14	8 sessions; 50 min; weekly	102 (50/52); 100.0%; participant randomization	Usual care	CDI	−0.73	−0.73^b^	^a^

a *No assessment at this time in the study*.

b *3-month follow-up assessment*.

c *6-month follow-up assessment*.

In the anxiety study sample (see Table 2), 13 studies used indicated prevention. Twelve studies were conducted in schools, one was community-based. Intervention group sizes varied from small groups of 5–12 adolescents in eight studies to large groups (school classes of 30 students) in three studies. Two studies did not report group size. The length of the interventions varied from 6 sessions of 45 min (total of 270 min) to 10 sessions of 120 min (total of 1,200 min). The control conditions were non-intervention groups in nine studies, waiting list groups in two studies, and active control conditions in two studies (guidance class and activity class). In three studies (Lowry-Webster et al., 2001; Barrett et al., 2005), school classes were given a universal preventive intervention and the data for at-risk adolescents were analyzed separately. In the other 10 studies, the intervention was presented to selected groups (i.e., elevated levels of anxiety).

**Table 2:** Summary of descriptive characteristics of anxiety prevention programs.

**Study: country**	**Summary of intervention**	**Type of prevention; school- or community-based**	**Sample**	**Mean age; age range**	**N per group**	**Characteristics of sessions**	**N (intervention group/control group); % females; randomization**	**Control group**	**Outcome measure**	**Effect size post-intervention**	**Effect size 3–6 months follow-up**	**Effect size 12 months follow-up**
Balle and Tortella-Feliu, 2010: Spain	Educational program about anxiety, the basics of some emotional regulation techniques, and gradual exposure to feared situations	Indicated; school-based	Children with high anxiety sensitivity, but no current mental health disorders or treatment	13.6; 11–17	10–12	6 sessions; 45 min; two times per week	92 (47/45); 61.0%; participant randomization	Waiting list	SCAS	0.09	−0.21^b^	^a^
Barrett et al., 2005 (group 1): Australia	FRIENDS program is based on CBT which assist children in learning important skills and techniques to cope with and manage anxiety and emotional distress	Indicated; school-based	High risk adolescents selected from a universal sample with elevated anxiety symptoms (SCAS > 32)	Unknown; 9–11	20–30	10 sessions (and two boosters); 45–60 min; weekly	47 (23/24); 76.6%; School randomization	Non-intervention	SCAS	−0.05	^a^	−0.56
Barrett et al., 2005 (group 2): Australia	FRIENDS program is based on CBT which assist children in learning important skills and techniques to cope with and manage anxiety and emotional distress	Indicated; school-based	High risk adolescents selected from a universal sample with elevated anxiety symptoms (SCAS > 32)	Unknown; 14–16	20–30	10 sessions (and two boosters); 45–60 min; weekly	19 (12/7); 73.7%; school randomization	Non-intervention	SCAS	0.24	^a^	−0.13
Dadds et al., 1997: Australia	Intervention, based on The Coping Koala, teaches children strategies for coping with anxiety and reinforces individual effort and change	Indicated; school-based	Adolescents identified by teachers as having anxiety disorders or showing elevated symptoms of anxiety (RCMAS ≥ 20)	9.4; 7–14	5–12	10 sessions; 60–120 min; weekly	128 (61/67); 75.8%; school randomization	Non-intervention	RCMAS	0.01	−0.05^b^	^a^
Gillham et al., 2012: US	Penn Resiliency Program is based on CBT and teaches participants the link between thoughts, feelings and behavior and skills for solving interpersonal problems and coping with stress	Indicated; school-based	Students with high levels of depression (on CDI and/or RADS-2)	Unknown; 10–15	Unknown	10 sessions; 90 min; weekly	266 (137/129); 48.0%; participant randomization	Non-intervention	RCMAS	−0.17	−0.10^b^	^a^
Gillham et al., 2006b: US	Penn Resiliency Program is based on CBT and teaches participants the link between thoughts, feelings and behavior and skills for solving interpersonal problems and coping with stress	Indicated; school-based	Students with the highest level of symptoms (on combined CDI and RCMAS Z-scores)	Unknown; 12–13	10–12	8 sessions; 90 min; weekly	44 (22/22); 70.5%; participant randomization	Non-intervention	RCMAS	−0.07	−0.63^b^	−0.80
Kiselica et al., 1994: US	The program based on Meichenbaum's stress inoculation training includes assertiveness training to provide participants with coping skills for dealing with external stressors	Indicated; school-based	Students with elevated anxiety symptoms (highest scores on STAI-A per class)	Unknown; 15	6	8 sessions; 60 min; weekly	48 (24/24); 45.8%; participant randomization	Active: guidance class	STAI	−0.74	^a^	^a^
Lock and Barrett, 2003: Australia	FRIENDS program is based on CBT which assist children in learning important skills and techniques to cope with and manage anxiety and emotional distress	Indicated; school-based	Adolescents with elevated anxiety symptoms (SCAS > 42)	Unknown; 9–16	Unknown	10 sessions (and two boosters); 70 min; weekly	66 (35/31); 75.8%; school randomization	Non-intervention	SCAS	0.20	^a^	0.10
Lowry-Webster et al., 2001: Australia	The FRIENDS program is CBT based and teaches children strategies for coping with anxiety and challenge situations	Indicated; school-based	Students with elevated symptoms of anxiety (SCAS > 42)	Unknown; 10–13	School classes	10 sessions; 60 min; weekly	108 (77/31); 52.9%; class randomization	Waiting list	SCAS	−0.80	^a^	^a^
Manassis et al., 2010: Canada	Feelings club is a CBT program focuses on recognizing and managing negative feelings and maladaptive thoughts using cognitive restructuring	Indicated; school-based	Children with elevated internalizing symptoms (MASC or CDI t > 60)	Unknown; 8–12	5–10	12 sessions; 60 min; weekly	148 (78/70); 43.2%; participant randomization	Active: activity class	MASC	−0.06	^a^	−0.06
Simon et al., 2011: Netherlands	The CBT based intervention teaches the children develop their own fear hierarchy, cognitive restructuring, task concentration training, and relaxation to decrease anxiety	Indicated; community-based	Children with elevated symptoms of anxiety (top 15% scores on SCARED)	10.1; 8–13	6–8	8 sessions; 90 min; weekly	114 (58/56); 57.0%; participant randomization	Non-intervention	SCARED	^a^	^a^	−0.13
Sportel et al., 2013: Netherlands	Program components were psycho-education, improving awareness of attentional focus control, cognitive restructuring, exposure, and relapse prevention	Indicated; school-based	Adolescents with elevated scores on social phobia or test anxiety (girls: RCADS > 10 or TAI > 43; boys: RCADS > 9 or TAI > 38) and low anxiety on ADIS-C	14.08; 13–15	3–10	10 sessions; 90 min; weekly	154 (84/70); 71.4%; school randomization	Non-intervention	RCADS	0.16	−0.41^c^	−0.13
Van Starrenburg et al., 2017: Netherlands	Dutch version of Coping Cat, reduced from 18 2- to 12 1-hourly sessions, teaches techniques such as relaxation, challenging thoughts, and problem-solving, and practicing with exposure	Indicated; school-based	Children with elevated symptoms of anxiety (SCAS > 1 SD above mean)	9.48; 7–13	7–9	12 sessions; 60 min; weekly	141 (66/75); 55.3%; participant randomization	Non-intervention	SCAS	−0.58	−0.64^b^	^a^

a *No assessment at this time in the study*.

b *3-month follow-up assessment*.

c *6-month follow-up assessment*.

### Outcome

**Figure 2:**
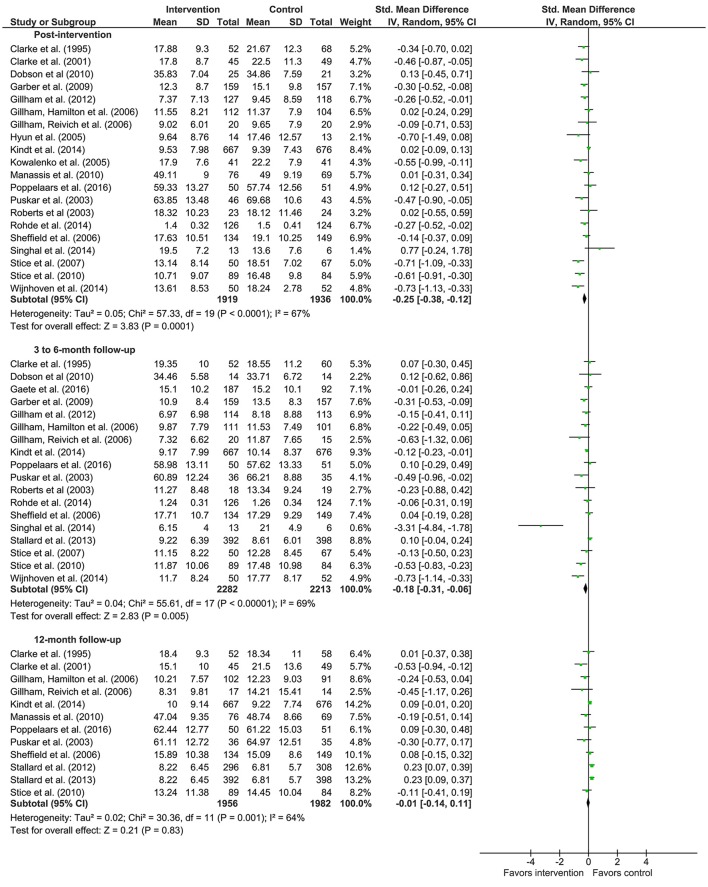
Forest plots of effects of depression prevention.

**Figure 3:**
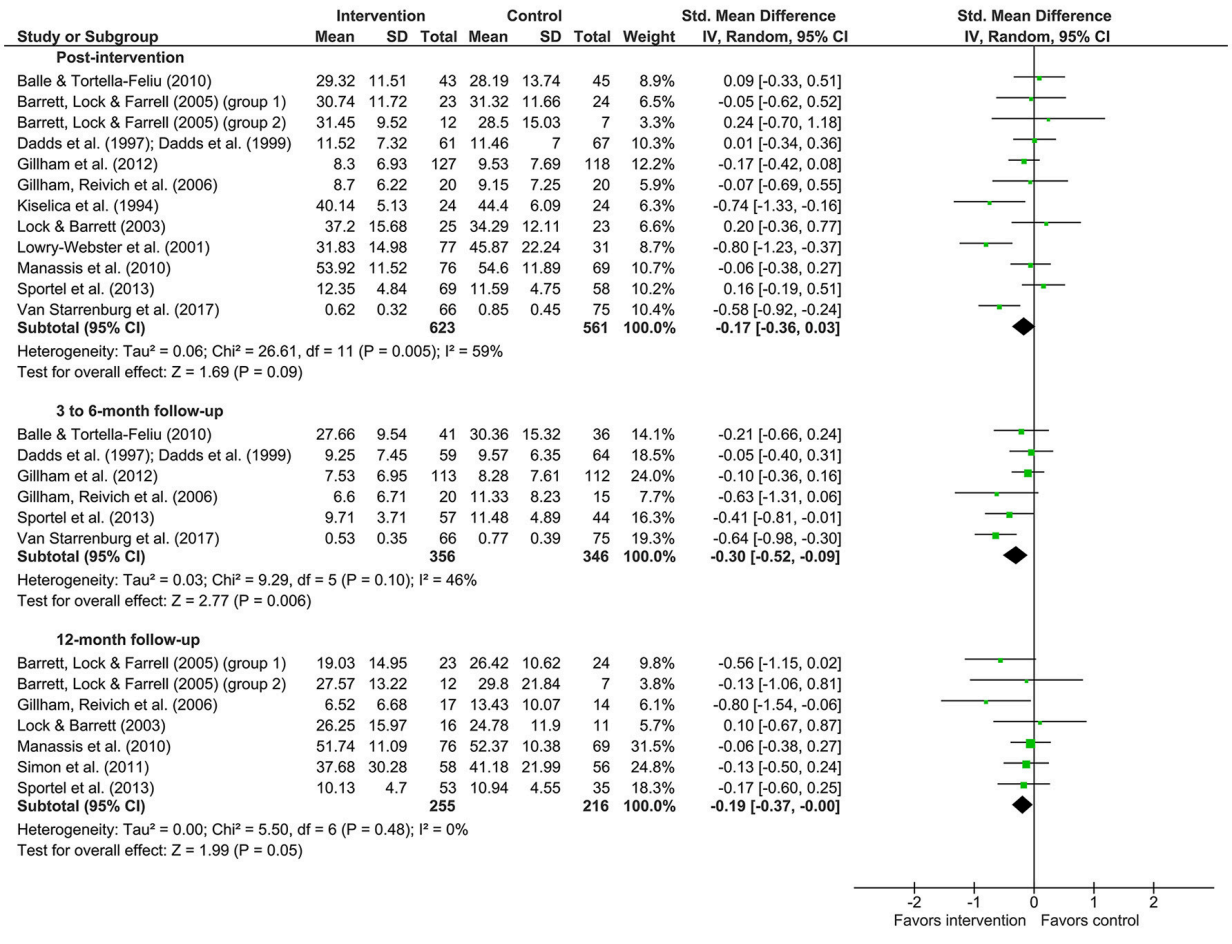
Forest plots of effects of anxiety prevention.

Meta-analyses were conducted on the data presented in Tables 1, 2. Means, standard deviations, number of participants, weight of the study and effect sizes are presented in Figure 2 for depression studies and Figure 3 for anxiety studies. Both are accompanied by a forest plot reporting effect sizes and confidence intervals per study (respectively square points and horizontal lines) and the pooled result for all studies (diamond). Measures of heterogeneity Tau^2^, Chi^2^, and *I*^2^ were calculated and presented in Figures 2, 3.

Concerning depression prevention, the meta-analysis showed that there was a small effect in improving depression symptoms post-intervention (20 studies; *d* = −0.25; 95% CI [−0.38, −0.12]). The meta-analytic *Z*-test showed a significant effect (*Z* = 3.83, *p* < 0.001). The heterogeneity test showed that the results of the various studies were moderately heterogeneous (Tau^2^ = 0.05, Chi^2^ = 57.33, *p* = <0.001; *I*^2^ = 67%).

There was no effect of depression prevention 3–6 months after the intervention (18 studies; *d* = −0.18; 95% CI [−0.31, −0.06]). The meta-analytic *Z*-test was significant (*Z* = 2.83, *p* = 0.005). The heterogeneity test revealed that the included studies were moderately heterogeneous (Tau^2^ = 0.04, Chi^2^ = 55.61, *p* < 0.001; *I*^2^ = 69%).

Depression prevention showed no effect (*d* = −0.01; 95% CI [−0.14, 0.11]) 12 months after the intervention (12 studies). The meta-analytic *Z*-test showed a non-significant effect (*Z* = 0.21, *p* = 0.83). The heterogeneity test showed that the various studies yielded moderate heterogeneous results (Tau^2^ = 0.02, Chi^2^ = 30.36, *p* = 0.001; *I*^2^ = 64%).

For anxiety prevention, the effect size (*d* = −0.17; 95% CI [−0.36,0.03]) for the post-intervention effects (12 studies) showed there was no effect. The meta-analytic *Z*-test showed a non-significant effect (*Z* = 1.69, *p* = 0.09). The heterogeneity test showed that the results of the included studies were moderately heterogeneous (Tau^2^ = 0.06, Chi^2^ = 26.61, *p* = 0.005; *I*^2^ = 59%).

Anxiety prevention showed a small effect (*d* = −0.30; 95% CI [−0.52, −0.09]) 3–6 months after the intervention (6 studies). The meta-analytic *Z*-test showed a significant effect (*Z* = 2.77, *p* = 0.006). The heterogeneity test revealed that the results of the included studies were low heterogeneous (Tau^2^ = 0.03, Chi^2^ = 9.29, *p* = 0.10; *I*^2^ = 46%).

Anxiety prevention 12 months after the intervention (7 studies) showed no effect (*d* = −0.19; 95% CI [−0.37, −0.00]). The meta-analytic *Z*-test showed a non-significant effect (*Z* = 1.99, *p* = 0.05). The heterogeneity test showed that the various studies yielded homogeneous results (Tau^2^ = 0.00, Chi^2^ = 5.50, *p* = 0.48; *I*^2^ = 0%).

## Discussion

This review described school-based and community-based prevention programs based on cognitive behavioral therapy with the primary goal of preventing depression and anxiety symptoms in adolescents at risk for developing these disorders. Furthermore, we determined the effectiveness in reducing symptoms of depression and anxiety directly after the intervention and at 3–6 and 12 months after the intervention.

The findings of our study revealed that selective and indicated depression prevention programs using techniques of cognitive behavioral therapy decrease symptoms of depression immediately after the intervention. However, the effects did not seem to last to 3–6 or 12 months after the intervention. In other words, the depression prevention generally decreases the symptoms of depression up to the end of the intervention. With this, the probability of the onset of a full-blown depressive disorder might slightly decrease. For anxiety prevention, we did not find evidence for effects directly after the intervention. However, there was an effect 3–6 to months after the intervention where anxiety prevention seems to reduce anxiety symptoms, although this effect disappeared 12 months after the intervention.

The findings of our study partially reflect the findings from previous research. They also reported the effects of depression prevention directly after the intervention (Horowitz and Garber, 2006; Calear and Christensen, 2010; Merry et al., 2012; Hetrick et al., 2016). In contrast to our study, they also reported effects during follow-ups. They reported, however, on a combination of several approaches of prevention in their studies, such as cognitive behavioral therapy, interpersonal therapy and mindfulness therapy, or focused on only school-based prevention programs, what might be an explanation for the different results. The studies that we included in the meta-analyses utilized a prevention program based on the principles of cognitive behavioral therapy, and therefore our findings can only be generalized to programs based on the cognitive behavioral approach. Cognitive behavioral therapy proved to be effective in treating depression in children and adolescents (Lewinsohn and Clarke, 1999; Michael and Crowley, 2002; Weisz et al., 2006). It is, therefore, likely that when these techniques are used in indicated and selective prevention of depression in adolescents, the results of these interventions are positive. Yet, effects of cognitive behavioral therapy based indicated and selective prevention programs seem to disappear after 3 months, and, therefore, improvements in effects on longer term are necessary before we can conclude that preventive interventions in high-risk populations, namely adolescents with substantial symptoms of depression, are meaningful for prevention of depression. In contrast to another review (Merry et al., 2012; Hetrick et al., 2016), we found no effects of depression prevention 3–6 and 12 months after the intervention. Our findings imply that depression prevention leads to short term symptoms reduction. The absence of an effect at 12 months after the intervention was also seen in the treatment of depression, where follow-ups of 1 year or more showed essentially no treatment effect (Weisz et al., 2006). There are several explanations for not finding an effect 12 months after the intervention. It is known that occasional long-term prevention sessions, so-called booster sessions, reduce the likelihood of relapse of depressive symptoms (Kroll et al., 1996). Without these booster sessions, the effectiveness of a prevention program might diminish during the following period. Most studies included in our meta-analyses did not use booster sessions following the preventive intervention, which might explain why depression prevention programs showed no effect 3–6 and 12 months after the intervention. Further, depression is known for its recurrent course and its fluctuations in level of depression symptoms (Judd et al., 1998; Kennedy and Paykel, 2004; Van Rijsbergen, 2014). This implies that in some adolescents depression symptoms might recur after some time, despite the preventive intervention they received, which is also advocated in different meta-analyses (Stockings et al., 2016). Especially when there is no change in the risk factors, the risk for depression remains high.

In contrast to other studies, we found that anxiety prevention programs did not show significant effects directly after the intervention or 12 months after the intervention (Neil and Christensen, 2009; Christensen et al., 2010), but there was an effect 3–6 months after the interventions. It is noteworthy that the effect sizes directly after the intervention and after 12 months in this study appear to be smaller compared to the effect sizes found in other reviews and meta-analyses. The main difference between our findings and those in other studies (Neil and Christensen, 2009; Christensen et al., 2010) concerns the inclusion criteria of the populations of the included studies. The previous reviews included studies on all types of anxiety prevention programs, namely studies with universal, selective, and indicated prevention programs. We, on the other hand, focused on adolescents at risk and included only studies with selective and indicated prevention programs using techniques of cognitive behavioral therapy. This implies that the severity of anxiety symptoms in our meta-analytic review was higher compared to other reviews, and to illustrate, adolescents in the studies we included reported elevated levels of anxiety up to clinical levels. The samples and the severity of their symptoms might play a role in the difference in outcome of this study and other review studies, more specifically, the level of symptoms might be too high in order for prevention programs to be effective direct after the intervention and for a longer period of time. Although the prevention programs that focused on anxiety used cognitive behavioral techniques, they were mainly based on social skills training (Dadds et al., 1997), relaxation exercises (Balle and Tortella-Feliu, 2010), and cognitive restructuring (Lock and Barrett, 2003). These techniques might be more effective in universal populations without symptoms of anxiety, as mentioned in earlier review studies (Neil and Christensen, 2009; Christensen et al., 2010), than in at-risk population, as in our study. The preventive interventions used in the studies that we reviewed may have lacked strong enough techniques for the prevention of anxiety in at-risk populations with symptoms up to clinical levels. The content of selective and indicated prevention programs for adolescents with elevated levels of anxiety that are almost reaching clinical levels of anxiety should presumably be more similar to the treatment of anxiety, as is done by Van Starrenburg et al. (2017). Therefore, we suggest that preventive interventions for anxiety in at-risk adolescents should not only use cognitive restructuring techniques, but should include exposure techniques and cognitive behavioral therapy as is done in treatment of anxiety disorders (Cartwright-Hatton et al., 2004; Compton et al., 2004; Rapee et al., 2009; Davis et al., 2011). This meta-analytic review focused on the selective and indicated prevention for depression and anxiety using cognitive behavioral therapy, whereas other studies included also universal prevention and other prevention techniques. This allowed us to draw conclusions about the cognitive behavioral therapy based prevention of depression and anxiety in adolescents with high risk for developing these disorders. We included only randomized controlled trials in our meta-analyses, which increased the internal validity of the studies in these meta-analyses. With this in mind, the results of the current studies can be interpreted with confidence. Limitations of the present study can be found in the studies included. The number of participants in some studies was low and differed largely in size across studies. We would like to indicate that the variations in sample size, in techniques used in the interventions, and in inclusion criteria resulted in lower heterogeneity between the anxiety prevention studies than between depression prevention studies. We think, however, that the similarities between the studies are larger than the methodological diversity. Another point of consideration about the lower effect sizes at longer follow-up, is the small number of studies with effects measured at longer follow-up periods. This causes a reduction of power with too few studies available to determine their true impact of selective and indicated prevention. Furthermore, the outcomes might have been influenced by the bias in selective reporting, as only studies with positive results were likely to be published and we selected only published studies. Also, some studies showed a lack of data, which we were not able to retrieve. Therefore, these studies were excluded from the meta-analysis, and this might have resulted in a bias outcome.

A small number of studies was included in the meta-analyses; therefore, potential moderating variables were not tested. Consequently, we cannot draw any conclusions about the influence of, for example, the size of the intervention groups, duration and intensity of the prevention programs, and the selection of the participants in the prevention programs, neither about sociodemographic characteristics, family history of depression, and level of elevated symptoms.

## Conclusion

This review presents evidence that cognitive behavioral therapy based prevention of depression in groups for high-risk adolescents is effective in the short term. These at-risk groups mostly have elevated levels of depression, and with these prevention programs their symptoms, and also the risk on a full-blown depressive disorder, reduce. For anxiety, cognitive behavioral therapy based prevention programs appear to be effective after 3–6 months, but this effect disappears after 12 months. The findings of the current meta-analytic review cautiously suggest depression and anxiety prevention programs based on CBT might have small effects on mental health of adolescents, although there should be improvements in effects before supporting the implementation of selective and indicated depression and anxiety prevention programs. It indicates that there is still much to be gained for prevention programs aimed at anxiety prevention. We focused on prevention of depression and anxiety in adolescents at risk using cognitive behavioral techniques. We, therefore, did not include universal prevention studies and studies using other techniques in our meta-analyses. Consequently, we could not compare universal prevention to targeted prevention, and we cannot conclude on about prevention programs based on for example interpersonal therapy or mindfulness therapy. Evaluations of the cost-effectiveness of depression and anxiety prevention should also be done, as they are currently lacking in the prevention literature. We suggest that, based on the similarities between treatment of depressive and anxiety disorder and the targeted prevention, the content of selective and indicated prevention programs for adolescents with subclinical depression and anxiety could profit from including techniques that have shown to be effective in the treatment of depression and anxiety.

## Author contributions

SR designed the manuscript, performed literature search, reviewed the literature, analyzed the data, and wrote the manuscript. DC, JJ, and RS contributed to the design of the manuscript, reviewed the literature, and revised the manuscript. All authors approved final version to be published.

### Conflict of interest statement

The authors declare that the research was conducted in the absence of any commercial or financial relationships that could be construed as a potential conflict of interest.
